# Endothelial mitochondria—less respiration, more integration

**DOI:** 10.1007/s00424-012-1085-z

**Published:** 2012-03-01

**Authors:** Lukas N. Groschner, Markus Waldeck-Weiermair, Roland Malli, Wolfgang F. Graier

**Affiliations:** Institute of Molecular Biology and Biochemistry, Center of Molecular Medicine, Medical University of Graz, Harrachgasse 21/III, 8010 Graz, Austria

**Keywords:** Endothelium, Mitochondria, Mitochondrial Ca^2+^, ROS, Atherosclerosis, Diabetes

## Abstract

Lining the inner surface of the circulatory system, the vascular endothelium accomplishes a vast variety of specialized functions. Even slight alterations of these functions are implicated in the development of certain cardiovascular diseases that represent major causes of morbidity and mortality in developed countries. Endothelial mitochondria are essential to the functional integrity of the endothelial cell as they integrate a wide range of cellular processes including Ca^2+^ handling, redox signaling and apoptosis, all of which are closely interrelated. Growing evidence supports the notion that impairment of mitochondrial signaling in the endothelium is an early event and a causative factor in the development of diseases such as atherosclerosis or diabetic complications. In this review, we want to outline the significance of mitochondria in both physiology and pathology of the vascular endothelium.

**Electronic supplementary material:**

The online version of this article (doi:10.1007/s00424-012-1085-z) contains supplementary material, which is available to authorized users.

## Introduction

As the blood vessel’s innermost layer, the vascular endothelium fulfills a great multitude of regulatory and sensory functions [23]. Impairment of any of these functions leads to distinct entities of cardiovascular diseases that collectively represent one of the major causes of overall morbidity and mortality in developed countries [103]. The functional intactness of the vascular endothelium is, therefore, of vital importance. The capability of mitochondria to dramatically interfere with endothelial function has just recently started to draw attention to this organelle.

The primary role of mitochondria is to produce and regulate the production of energy-rich molecules such as adenosine triphosphate (ATP), via aerobic respiration. At first impression, these cellular power plants might not seem to be of particular importance to the endothelial cell, a type of cell that usually covers over two-thirds of its energy demands by anaerobic glycolysis [32, 33, 118], but at a second glance, mitochondria turn out to be essential to endothelial function in many ways and are far more than just a source of ATP.

Mitochondria constitute multifunctional organelles that are able to specifically regulate the signaling of cellular messengers such as Ca^2+^ and reactive oxygen species (ROS) within cells. In addition, mitochondria have been recognized to determine the fate of cells by controlling apoptosis, the process of programmed cell death. In this review, we intend to take a closer look at these specific functions and possible dysfunctions of mitochondria in the vascular endothelium.

## Mitochondrial contribution to endothelial physiology

### Endothelial cell physiology

Endothelial cells line the inner surface of blood vessels and establish an active barrier between solid tissues and circulating blood. Over the past few decades, this delicate cellular monolayer has emerged as a highly specialized tissue that by far exceeds the sole function of forming a passive physical interface [23]. Endothelial cells actively regulate an enormous variety of physiological processes and thereby control vascular tone and permeability, inflammatory responses, leucocyte trafficking, platelet adhesion and hemostasis, angiogenesis and wound healing as well as the exchange of metabolites between blood and surrounding tissues (for a review see Ref. [23]). The vascular endothelium also interacts with other cell types such as vascular smooth muscle cells, leukocytes, platelets, stem cells, pericytes, cardiomyocytes, mesangial cells of the kidney and many more mainly through the production of signaling and adhesion molecules [23]. For example, nitric oxide (NO), prostacyclin, hyperpolarizing factor and endothelin, all generated in the endothelium, directly modulate vascular smooth muscle contractility [98]. Secretion of von Willebrand factor causes platelet adhesion, while selectins, produced by the endothelium, are responsible for the recruitment of leukocytes during inflammation [23]. Hence, even slight dysfunction of the endothelium can also compromise the function of each of these cell types leading to inadequate vasoconstriction, leukocyte infiltration, coagulation, permeability and increased proliferation or apoptosis [23], all of which are key features found in a wide range of diseases that represent the leading causes of death in the western world [63].

Endothelial cells per se are quite heterogeneous and various phenotypes meet the particular demand of the individual tissue and/or organ [47, 48]. Some differences in endothelial architecture such as permeability and fenestration can be easily deduced from differences in the specific function of the organs. Others are not as obvious but become very clear in diseases like atherosclerosis, vasculitis or even tumor metastasis that often manifest at preferential sites within the circulatory system [23]. Endothelial cells of different origins might even show diversity in mitochondrial physiology such as DNA repair ability [33, 58].

### Mitochondria: structure and function

The mitochondrion exhibits unique architecture, comprising of an inner membrane encased by an outer membrane dividing it into two distinct compartments [95]. The outer membrane, separating the intermembranous space from the cytosol, is permeable to most sorts of ions and small molecules via the voltage-dependent anion-selective channel (VDAC) that has been found, at least in liposomal preparations, to be activated by high Ca^2+^ [8]. Ion flux through the inner mitochondrial membrane (IMM), however, is tightly regulated by the activity of selective mitochondrial ion channels and exchangers [93]. Along with these ion shuttling proteins, the IMM, which forms out cristae in order to expand its surface, houses a multitude of multiheteromeric protein complexes that account for electron transport and oxidative phosphorylation (OXPHOS) [125].

**Fig. 1:**
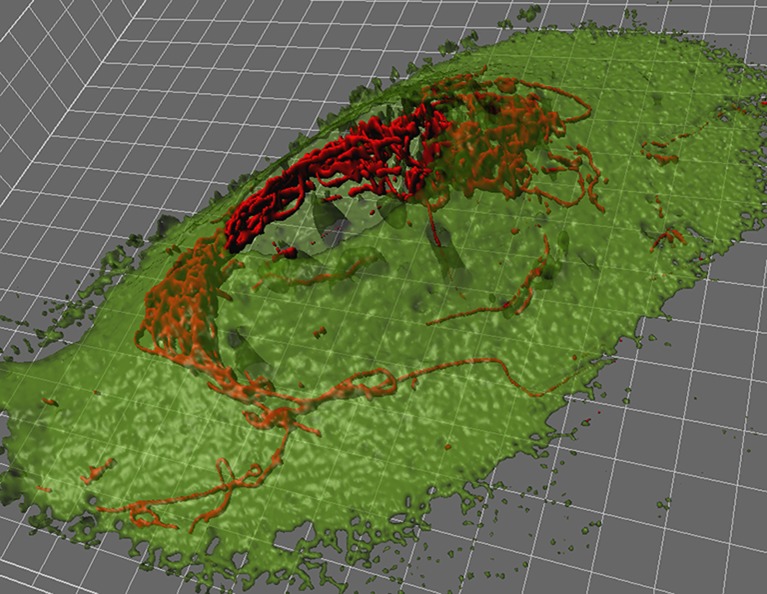
3D-rendered micrograph visualizing the morphology of mitochondria (*red*) in the human umbilical vein endothelial cell line EA.hy926

The microscopic anatomy of mitochondria within living cells is fascinating by various means. Mitochondria are not at all static organelles but move and continuously change their appearance by fission, fusion and branching. The organelles can appear as highly interconnected tubular structures forming a complex network but also as smaller single beads or rods within one given cell (Fig. 1). Fast remodeling of the overall morphology of mitochondria is believed to be fundamental for maintaining intact mitochondrial DNA (mtDNA), metabolic function and signaling of these organelles [95, 108, 154]. The dynamics of individual mitochondria within one given cell can be very diverse (Supplementary movie 1), probably depending on the connection to certain motor proteins [135] and the cytoskeleton. This heterogeneity in the movements of single mitochondria also impacts mitochondrial fusion–fission dynamics [87] and might reflect differences in metabolic activity and signaling of individual mitochondria.

Mitochondria exhibit tissue-specific characteristics including their capacity of ATP production [32, 33, 118], their number and distribution [107], and also, mechanisms of Ca^2+^ handling appear to vary between cell types [9, 34, 39, 72]. In endothelial cells, mitochondria comprise less than 5% of total cellular volume (Fig. 1) compared to around 28% in hepatocytes [13]. That and the point that endothelial cells do not necessarily depend on OXPHOS in order to produce ATP [118] have led to the fact that the role of mitochondria in endothelial physiology and pathology has been underestimated for a long time.

For many years, isolating mitochondria from fresh tissue is a well-established technique to investigate specific processes in a suspension of mitochondria [81]. However, in vitro experiments are restricted to the evaluation of a few basic functions that are preserved in purified mitochondria such as ATP production and oxygen consumption. As mentioned above, these functions might not be among the most prominent duties of mitochondria in the endothelium. Since the development of genetically encoded sensors that enable the assessment of mitochondrial physiology in living cells, mitochondria have emerged as signaling organelles with a wide range of functions reaching far beyond the mere task of ATP synthesis.

Mitochondria vividly interact with other organelles and contribute substantially to endothelial Ca^2+^ signaling (reviewed in Ref. [51]), ROS production, apoptosis, lipid metabolism and autophagy. Even endothelial NO production is influenced by mitochondrial Ca^2+^ handling [36].

### Oxidative phosphorylation (OXPHOS)

OXPHOS, the aerobic production of ATP by mitochondria, requires a stepwise oxidation of electron donors reduced through catabolism of fuels comprising lipids, amino acids and, most importantly to the endothelial cell, carbohydrates. This depends on the orchestrated action of huge multiheteromeric protein complexes anchored to the IMM and encoded by both nuclear and mtDNA, commonly referred to as the mitochondrial electron transport chain (ETC) [125].

Under most conditions, endothelial ATP supplies can be covered by anaerobic metabolism of d-glucose, which renders endothelial OXPHOS an oxygen-consuming luxury and rather counterproductive in supplying adjacent tissues with sufficient oxygen [49]. However, low basal rates of oxygen consumption, together with NO, might help the endothelium in maintaining an adequate oxygen gradient around blood vessels.

NO, produced by endothelial NO synthase (eNOS; NOS-III), seems to have a central role in sensing O_2_ concentrations and controlling respiration as it binds and inhibits complex IV of the ETC (cytochrome c oxidase) in an O_2_-dependent manner. This inhibition is reversible and inversely correlates with ambient O_2_ concentrations [100]. Activation or inhibition of eNOS has been found to cause corresponding alterations in mitochondrial oxygen consumption [25]. This means that when local O_2_ concentrations fall, NO produced by eNOS [153] causes vasodilation but also restricts OXPHOS of endothelial and perivascular cells, thus, allowing O_2_ to diffuse deeper into the tissue.

OXPHOS is the main source of ROS [102] and even though the importance of OXPHOS to endothelial cells remains questionable, ROS of mitochondrial origin are discussed as key factors in the pathophysiology of cardiovascular diseases [18, 89, 105]. A disproportionately high supply of metabolites and, thus, also electron donors, is believed to overburden the mitochondrial ETC, thereby causing increased leakage of unpaired electrons at preferential sites to O_2_ [89]. However, recent studies describe enhanced endothelial ROS formation not only under hyper- but also under hypoglycemic conditions [149], which might raise some doubts about ROS being actually the initial instigator of endothelial dysfunction.

### Mitochondrial Ca^2+^ handling

Calcium ions stand out among other cations due to their ability to act as second messengers and thereby modify an impressive range of cellular processes. This can be achieved by reversible binding of Ca^2+^ to different Ca^2+^-binding domains within a variety of signaling proteins. Ca^2+^-induced conformational changes of these specific proteins lead to alterations in enzyme activity, subcellular localization and other properties [51] that govern endothelial function. Considering the abundance of cytosolic Ca^2+^-binding proteins and the basal intracellular Ca^2+^ concentration being relatively low with around 100 nM, it becomes obvious that the diffusion coefficient of ionic Ca^2+^ in the cytosol is vanishingly small, making it a basic necessity for the cell to form out Ca^2+^ signaling networks that help in conducting the sophisticated interplay of subcellular Ca^2+^ handling. For a long time, mitochondria were considered to act as a passive sink for Ca^2+^, storing it within the matrix without any further activity, but over the last few years, mitochondria have been found to actively take part in cellular Ca^2+^ homeostasis. Although the main intracellular source of Ca^2+^ is represented by the endoplasmic reticulum (ER), mitochondria are estimated to account for at least 25% of total Ca^2+^ in endothelial cells [152] depending on the state of cell activation. Together mitochondria and the ER establish junctions to cooperate in the propagation of Ca^2+^ signals [64, 121].

It is of crucial importance to most types of cells that the Ca^2+^ concentration in the ER ([Ca^2+^]_ER_) does not drop below a certain level, since the activity of ER chaperones such as calreticulin and calnexin strictly depends on free Ca^2+^ [26]. These lectin chaperones are responsible for proper assembly and quality control of glycoproteins [26, 109]. Hence, a persistent decrease in [Ca^2+^]_ER_ causes protein misfolding, accumulation of these proteins and activation of the unfolded protein response and ER stress pathways [26], which have been found to contribute to endothelial pathology [24]. In order to avoid such a scenario, refilling of ER Ca^2+^ stores is accomplished by a highly efficient machinery that guarantees adequate ER Ca^2+^ content in the endothelium even under prolonged stimulation with inositol 1,4,5-triphosphate (IP_3_)-generating agonists like histamine [92]. Endothelial mitochondria are key components of this machinery as they readily take up Ca^2+^ at the inner mouth of capacitative Ca^2+^ entry/I_CRAC_ channels which, together with the clustering of stromal interaction molecule 1 (STIM1), are highly sensitive to Ca^2+^ gradients in their vicinity [94]. By actively decreasing local cytosolic Ca^2+^ concentrations, mitochondria are able to promote the formation of STIM1 clusters and keep I_CRAC_ channels in the open conformation, thus, contributing to the maintenance of store-operated Ca^2+^ entry (SOCE) in the endothelium [91, 92]. A similar phenomenon has been described in T lymphocytes [69]. In contrast, it has been shown that the ER Ca^2+^ content in adrenal glomerulosa cells is not preserved during prolonged cell stimulation with an IP_3_-generating agonist [4], despite a distinct activation of SOCE [122].

Nevertheless, the simultaneous increase in mitochondrial Ca^2+^ concentration ([Ca^2+^]_mito_) enhances mitochondrial ATP production, which is essential for Ca^2+^ uptake into the ER through sarco/ER Ca^2+^-ATPase (SERCA) [112].

Unlike the ER, mitochondria do not store Ca^2+^ for longer time periods, but rather help in funneling Ca^2+^ to the ER for fast replenishment [92]. A similar modulation of ER refilling by mitochondria can be observed in HeLa cells [2]. This also explains the short duration of mitochondrial Ca^2+^ elevations that is mainly influenced by a balance between the mitochondrial Ca^2+^ uniporter(s) (MCU) and the activity of the electroneutral mitochondrial Na^+^/Ca^2+^ exchanger (NCX) [91]. In endothelial cells, inhibition of NCX by either pharmacological compounds [91] or treatment with ROS [73] causes prolonged mitochondrial Ca^2+^ elevation and, in consequence, also insufficient Ca^2+^ refilling of the ER during continuous stimulation, the latter of which has been generally neglected so far. This impairment of cellular Ca^2+^ signaling could conceivably affect endothelial function and, in the worst case, both excess mitochondrial Ca^2+^ and ER stress could trigger apoptotic pathways. All of these are major culprits in the initiation of cardiovascular diseases [24, 158], but still the molecular identities of many proteins involved in endothelial Ca^2+^ handling are yet to be discovered.

In general, Ca^2+^ transport across the IMM is tightly regulated by a set of proteins that include more or less Ca^2+^ selective ion channels (MCU, UCP2/3, mRyR), exchangers (Letm1, NCLX) and regulatory proteins (MICU1) as well as the nonselective mitochondrial permeability transition pore (mPTP) [51]. In respiring mitochondria, Ca^2+^ flux through the IMM is mainly driven by the enormous IMM potential (Δ*Ψ*_*m*_) of around −180 mV that establishes a strong electromotive driving force for Ca^2+^ to enter the mitochondria via the so-called MCU. This channel exhibits high selectivity for, but rather low affinity to, Ca^2+^ ions [76]. The MCU’s *K*_*D*_ of 10–15 μM, originally described in studies on isolated cardiac mitochondria [128], does not match the actual calcium signals observed in living cells where cytosolic Ca^2+^ concentrations are far lower, prompting the idea of cellular microdomains with high local concentrations of calcium [60]. In endothelial cells, such foci could conceivably be found at junctions between mitochondria and the ER and close to I_CRAC_ channels in the subplasmalemmal space.

**Fig. 2:**
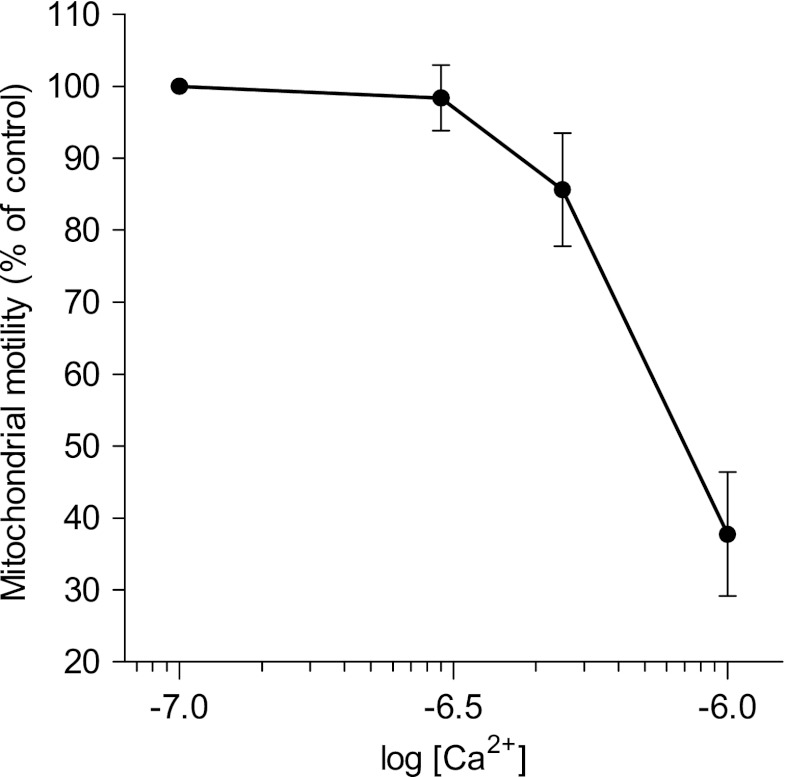
Mitochondrial motility inversely correlates with the Ca^2+^ concentration ([Ca^2+^]) in ionomycin-permeabilized endothelial cells. Cells expressing mtDsRed were incubated in a Ca^2+^-free buffer containing 10 μM ionomycin and 5 mM EGTA (0 Ca^2+^) for 3 min. Confocal images (Δ*t* = 1 s) of mtDsRed fluorescence were recorded under conditions of 100 nM (*n* = 5), 300 nM (*n* = 5), 500 nM (*n* = 5) or 1000 nM (*n* = 5) clamped free Ca^2+^ for 3 min. Mean mitochondrial motility for each concentration was calculated by the number of mtDSRed-positive pixels changing *x*–*y* location in between time points (Δ*t* = 10 s). Values indicate the percentage of mitochondrial motility relative to the mitochondrial motility under clamped Ca^2+^-free conditions and are represented as means ± SEM. This analysis did not allow discrimination between oscillatory and unidirectional movements

In line with this hypothesis is the finding that mitochondrial motility is highly sensitive to cytosolic Ca^2+^. This phenomenon can be observed in various cell types including endothelial cells (Fig. 2, Supplementary movie 2). Recent studies in cell lines derived from neuronal and cardiac tissues have identified the Miro–Milton protein complex to account for the Ca^2+^ sensitivity of mitochondrial dynamics [86]. Containing two EF hands, Miro has been shown to serve as a Ca^2+^ sensor causing mitochondria to retain at sites of high cytosolic Ca^2+^ concentrations [124] where they seem to participate in buffering Ca^2+^ [91].

Anchoring mitochondria permanently to the inner plasma membrane of endothelial cells decreased mitochondrial Ca^2+^ uptake in response to SOCE [104]; hence, one can assume that mitochondria actively move to sites of Ca^2+^ entry. Local buffering of entering Ca^2+^ by mitochondria yields global mitochondrial Ca^2+^ transients during SOCE in the endothelium, although only 10% of mitochondria are found in close vicinity to subplasmalemmal STIM1 clusters [104]. Due to the morphological and functional heterogeneity of mitochondria (Fig. 1) [15, 27], locally sequestered Ca^2+^ obviously cannot spread out over the entire mitochondrial network. Consequently, other mechanisms accounting for the propagation of mitochondrial Ca^2+^ transients in the endothelium might exist including Ca^2+^ cycling between mitochondria and cytosol, as it has been proposed for astrocytes [14].

This would also favor the existence of variable mechanisms of mitochondrial calcium sequestration or some kind of molecular switch that might adjust the Ca^2+^ affinity of the MCU to different concentrations of cytosolic Ca^2+^. In endothelial cells uncoupling protein 3 has been proposed to act as such [145]. In fact, there are several proteins that have been described to contribute to the phenomenon of mitochondrial Ca^2+^ uniport including uncoupling proteins 2 and 3 (UCP2/3) [137, 138]. Just recently, after the identification of mitochondrial calcium uptake 1 (MICU1) as a regulatory subunit [114], the actual pore-forming component of the MCU was discovered [9, 34] in experiments using HeLa and HEK-293 cells as well as isolated mouse liver mitochondria. Nevertheless, there is data pointing to more than just one exclusive way of mitochondrial Ca^2+^ uptake depending on the cell type as well as the source of Ca^2+^ [72, 146, 147]. Electrophysiological experiments have provided evidence for at least two distinct calcium currents in the IMM of human cardiomyocytes [99] and HeLa cells and three in endothelial cells [72]. In the endothelium, UCP2/3 seem to be especially important for the sequestration of Ca^2+^ released from the ER but not for Ca^2+^ uptake during SOCE [138, 147]. In order to fully grasp the differential regulation of these mechanisms, it will be a basic necessity to employ a great variety of experimental approaches and sophisticated techniques [72].

When Ca^2+^ passes through the mitochondrial matrix, it leaves its traces influencing some key features of mitochondrial physiology that depend on the presence of free Ca^2+^. The activity of some mitochondrial dehydrogenases has been shown to require mitochondrial free Ca^2+^ [62]. This Ca^2+^-dependent regulation of mitochondrial metabolism is cell-specific and varies with the function of the cell [117]. Ca^2+^ can both directly activate enzymes of the TCA cycle such as isocitrate dehydrogenase and 2-oxoglutarate dehydrogenase [96] and increase enzyme activity of pyruvate dehydrogenase via Ca^2+^-dependent dephosphorylation [37]. In that way, Ca^2+^ exerts its sustained modulatory effects on mitochondrial ATP generation [74]. Cytosolic Ca^2+^ signals being transferred into mitochondria probably help the cell to keep up with increasing energy demands under activated conditions. However, this feedback mechanism also depends on the source of Ca^2+^ and the type of cell [123]. The relevance of this pathway in the highly glycolytic endothelial cell still remains unclear, but it seems possible that cell activation also requires the recruitment of additional ATP sources, which are negligible during resting states. Another condition that accelerates mitochondrial metabolism and thereby also generation of free radicals in the endothelium is hyperglycemia [18]. Restriction of OXPHOS by inhibiting MCU might provide future treatment options for diabetic complications.

NO, an integral player in cardiovascular physiology, regulates vascular smooth muscle contraction, ion channel activity, apoptosis and also oxygen consumption [12, 25, 143] (reviewed in Ref. [46]). In fact, NO has been shown to reduce Δ*Ψ*_*m*_ [136] possibly by reversibly inhibiting complex IV of the mitochondrial ETC [49] and modulating mPTP opening [17], both of which could either be responsible for or a consequence of an inhibition of mitochondrial Ca^2+^ uptake by NO [35]. Notably, a mitochondrial isoform of NO synthase is activated by mitochondrial free Ca^2+^, representing a negative feedback mechanism that has been proposed to protect against mitochondrial Ca^2+^ overload during exposure to high concentrations of NO [36].

### ROS production and redox signaling

Mitochondria represent one, if not the major, source of ROS within most cell types. ROS are reactive compounds that can originate as a by-product of OXPHOS, mainly as a result of leakage of single unpaired electrons from the ETC reducing molecular oxygen to generate superoxide anions. These superoxide radicals are readily converted into other ROS including hydrogen peroxide, peroxynitrite and hydroxyl radicals [102]. At low levels, ROS are believed to play an essential role in vascular signaling processes by regulating the activity of signaling proteins, enzymes and ion channels in endothelial cells [116, 132]. NO, for instance, the endothelium-derived relaxing factor, being one of the most studied cellular signaling molecules, is a free radical itself. Yet NO is essential to endothelial function as it mediates vasodilation along with numerous other physiological processes [46]. Other targets of mitochondrial ROS signaling in endothelial as well as surrounding smooth muscle cells are proliferation, hypertrophy and apoptosis [59].

On the other hand, an excess of ROS, if not detoxified, can cause damage to macromolecules including DNA, proteins and lipids, commonly referred to as oxidative stress. Alterations in protein function and accumulation of modified proteins as well as DNA strand breaks and mutations, over time lead to cellular dysfunction and contribute to endothelial damage in diseases like atherosclerosis and diabetes [18]. Under these conditions, mitochondrial ROS clearly contribute to the initiation of inflammatory pathways such as NF-κB activation in the endothelial cell that, in turn, lead to the recruitment of leucocytes to the vascular intima [111]. The subsequent oxidative burst provoked by activated neutrophils is orders of magnitude higher than endothelial ROS generation and usually results in sustained damage to the endothelium [139].

The close connection between cellular ROS and Ca^2+^ signaling is reflected by the impact of ROS treatment on mitochondrial Ca^2+^ handling in endothelial cells. Oxidative stress provoked by either a H_2_O_2_ bolus or a combination of hypoxanthine and xanthine oxidase potentiates mitochondrial Ca^2+^ signals by inhibiting NCX [73]. This mechanism seems to work in both ways, since mitochondrial Ca^2+^ transients can also greatly increase the generation of free radicals [20]. One example for the cooperation of mitochondrial Ca^2+^ and ROS in endothelial cell signaling shall be depicted in the form of their function in mechanotransduction. As mitochondria are anchored to the cytoskeleton [85], they can sense shear stress and increase ROS production in response [1]. A clear attenuation of shear stress-induced ROS signaling and consecutive expression of adhesion molecules were seen after selective inhibition of mitochondrial ROS production, while inhibition of other ROS sources had no effect [1]. Another report describes that increased blood pressure in the pulmonary circulation leads to Ca^2+^ oscillations in the cytosol of endothelial cells, which are transferred to the mitochondria where they promote ROS production and thereby also exocytosis of P-selectin [71].

## Mitochondrial contribution to endothelial pathology

### Controlled apoptosis in endothelial cell function

The development of new blood vessels as well as the regression of preexisting ones depend on the tightly regulated balance between proliferation and the controlled death of endothelial cells [90]. Mitochondria have a critical function in triggering this enzymatic cascade of self-destruction by releasing a set of proteins into the cytosol [142]. This happens in response to proapoptotic stimuli that can originate either from inside the cell itself or from outside by activation of death receptors. A lot of in vitro studies suggest that endothelial cell apoptosis might play a special role in the pathophysiology of micro- and macroangiopathy under certain conditions like diabetes or hyperlipidemia [42, 144]. Unlike necrosis, apoptosis follows a stereotypical cascade of events that can be influenced pharmacologically, which is why the identification of the exact molecular mechanisms leading to endothelial cell death is that important.

Mitochondrial pathways of apoptosis usually involve the permeabilization of the OMM leading to a release of cytochrome c from the intermembranous space into the cytosol where it associates with other proteins including caspase 9 to form the so-called apoptosome [61]. In many but not in all cases, this is accompanied by the formation mPTP, a large unselective pore spanning both IMM and OMM that allows ions and small molecules to permeate, thus, causing a dissipation of Δ*Ψ*_*m*_ [22]. Inhibition of mPTP opening by treatment with cyclosporine A seems to prevent cytochrome c release and apoptosis in human endothelial cells [148], although in other cell types release of cytochrome c has been reported to occur upstream of mPTP opening [16].

Mitochondrial Ca^2+^ overload is one of the factors that independently cause opening of mPTP. During sustained elevations of cytosolic Ca^2+^, observed in multiple pathological conditions of the endothelium [134, 144], free Ca^2+^ inside the mitochondrial matrix progressively rises. In the presence of cyclophilin D, this induces the formation of mPTP [7] that is kept open as long as the cytosolic Ca^2+^ elevation persists. The pore allows solutes to freely diffuse into the mitochondrial matrix leading to mitochondrial swelling and rupture of the OMM followed by release of proapoptotic factors [61].

Another mitochondrial pathway of apoptosis is characterized by the release of apoptosis-inducing factor (AIF), a protein involved not only in apoptosis, but also in free radical scavenging and OXPHOS. The translocation of AIF from the IMM to the nucleus requires permeabilization of the OMM and removal of the inner membrane anchoring segment, the latter of which is achieved by activation of the Ca^2+^-dependent protease calpain I [75]. This specific pathway of apoptosis was reported to be implicated in endothelial cell loss in diabetic retinopathy [83].

### Atherosclerosis

Even though mtDNA encodes only 13 of all mitochondrial proteins, there are associations of mtDNA damage [28] as well as certain mtDNA haplogroups with coronary artery disease and diabetic retinopathy [102]. Although some of these associations are not confirmed by other studies [51], they still highlight the importance of mitochondria in cardiovascular disease. MtDNA is more prone to ROS-induced damage than nuclear DNA because firstly, it is in close vicinity to the ETC as the major source of ROS, and secondly, it lacks many repair mechanisms of nuclear DNA [29, 155].

In view of these reports, the question arises, if alterations of mtDNA are just an indicator of oxidative damage or if they also play a causative role in the process of atherogenesis possibly preceding the formation of atherosclerotic lesions. In this respect, the fact that not only acquired but also connate mtDNA mutations predispose to cardiovascular disease already points to mtDNA damage occurring at an early stage of atherosclerosis, which is further supported by investigations in apolipoprotein E knockout (apoE^−/−^) mice [5]. What is/are the initiating factor(s) causing mtDNA damage? Mitochondrial ROS production itself [45] as well as exogenously administered ROS [6] have been shown to cause mtDNA damage, impaired mitochondrial protein synthesis and ATP production, thus creating a vicious circle of increasing oxidant generation and decreasing mitochondrial function [59]. For example, a complex I deficiency of the respiratory chain, which can be caused by mutations in any of the genes encoding a subunit of complex I, leads to excessive production of superoxide [115].

While in diabetes it is mainly hyperglycemia that is believed to account for the initial excess in mitochondrial ROS (see “Diabetes”), another important etiologic factor in the development of atherosclerotic lesions is the oxidative modification of low density lipoprotein (LDL), which further promotes inflammatory responses of the endothelium [133]. It has been convincingly demonstrated that certain lipid oxidation products specifically accumulate in endothelial mitochondria [80] where they seem to increase mitochondrial ROS production [160]. Apart from causing DNA damage, inflammatory responses and a decline in mitochondrial function, oxidative stress also leads to increased apoptosis [22, 78] and so does oxidized LDL (oxLDL) in the endothelium [38, 44, 65]. Recently, a couple of substances have been discovered that protect endothelial cells against oxLDL-related apoptosis. For example, exogenous administration of humanin, a peptide found in endothelial mitochondria, can partly abrogate the effects of oxLDL [3]. Another study has shown that apoptosis, oxidative stress and progression of atherosclerosis in apoE^−/−^ mice can be positively influenced by treatment with humanin [106]. Further protective compounds include the natural phenol resveratrol, and its analogs that are potent inducers of sirtuin gene expression [159]. Some of these sirtuins, namely SIRT3, SIRT4 and SIRT5 are found exclusively in the mitochondrial matrix where SIRT3 acts as NAD^+^-dependent deacetylase, thereby directly influencing energy metabolism. Other sirtuins that are not located in the mitochondrion such as SIRT1 still have substantial influence on mitochondrial biogenesis in the endothelium [30].

The phenomenon of oxLDL-induced apoptosis of endothelial cells in vitro, which was already described to depend on Ca^2+^ [44], has more recently been found to involve both caspase-dependent and caspase-independent mitochondrial pathways [21, 144]. Noteworthy, cyclosporine A, which has properties of inhibiting mPTP [157], has been shown to reduce oxLDL-associated atherosclerosis [40] by preventing the release of cytochrome c from mitochondria [148].

Interestingly, in cultured endothelial cells, the oxidation of LDL itself correlates with mitochondrial superoxide production and can be stopped by inhibition or uncoupling of the ETC [88] pointing to a vicious circle that ultimately culminates in vascular pathologies.

Over the past few years, more and more experimental evidence has accumulated that links mitochondrial dysfunction to atherosclerosis (reviewed in more detail in Ref. [89]).

### Diabetes

Diabetes mellitus is characterized by an elevation of blood glucose caused by insulin resistance and/or deficiency, in most cases accompanied by hyperlipidemia. Due to their direct exposure to the blood and all its components, endothelial cells obviously represent the primary target of both hyperglycemia as well as hyperlipidemia. Under these conditions, the endothelium is practically overwhelmed by pathologically high levels of d-glucose.

Since M. Brownlee proposed a unifying mechanism of diabetic complications 10 years ago, endothelial mitochondria have become a new focus in diabetes research [18, 19]. According to Brownlee’s hypothesis, an excess of d-glucose in the endothelial cell is metabolized via glycolysis yielding pyruvate that enters the mitochondria and feeds into the TCA cycle providing an increasing amount of reducing equivalents [151] and, thus, an increased flux of electrons through the respiratory chain. As a consequence, mitochondrial superoxide production is boosted, causing oxidative damage to all sorts of molecules including nuclear DNA. Subsequently, mechanisms of DNA repair such as poly(ADP-ribose) polymerase (PARP) are activated, which further causes collateral damage by decreasing the activity of GAPDH, a central enzyme of glycolysis [41]. The accumulation of glycolytic intermediates then promotes the different downstream pathways of hyperglycemic damage [18, 105].

With 30 mM, the d-glucose concentrations used in these studies to mimic diabetic conditions were rather high compared with the situation in humans. Considering that even patients with slightly impaired glucose tolerance already face significantly worse prognosis [131], it is tempting to speculate that there might be additional factors eliciting endothelial dysfunction, possibly including mitochondrial dysfunction and ER stress as outlined above.

High d-glucose also provokes alterations in mitochondrial dynamics [110] and Ca^2+^ signaling [56, 150] that might even precede the ROS burst itself [52, 54] and, thus, represent attractive targets for future therapeutic interventions [79]. Enhanced fragmentation of mitochondria is at least partly caused by increased expression and activity of proteins mediating mitochondrial fission such as fission-1 protein (Fis1) and dynamin related protein-1 (Drp1) [130]. Silencing of either of the corresponding genes reduces mitochondrial ROS production and restores eNOS activity under hyperglycemic conditions [130, 156].

**Fig. 3:**
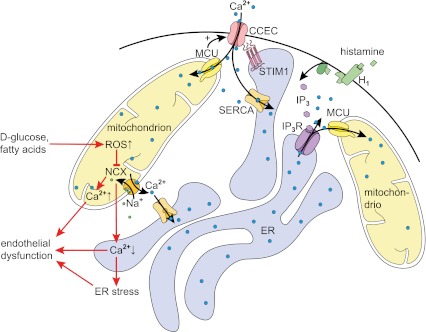
Schematic illustration of *trans*-organelle Ca^2+^ signaling in endothelial cells upon IP_3_-mediated intracellular Ca^2+^ release and SOCE. *Black arrows* indicate Ca^2+^ movements under physiological conditions in response to endothelial cell stimulation with, e.g., histamine. *Red lines* highlight pathological processes under substrate overload that cause excessive mitochondrial ROS production and, in turn, alter organelle Ca^2+^ homeostasis leading to endothelial cell dysfunction. *CCECs* capacitative Ca^2+^ entry channels, *ER* endoplasmic reticulum (*blue*), *H*_*1*_ histamine H_1_ receptor, *IP*_*3*_ inositol 1,4,5-triphosphate, *IP*_*3*_ *R* IP_3_ receptor, *MCU* mitochondrial Ca^2+^ uniporter, *NCX* mitochondrial Na^+^/Ca^2+^ exchanger, *SERCA* sarco/endoplasmic reticulum Ca^2+^-ATPase, *STIM1* stromal interaction molecule 1

Mitochondrial fission during treatment with high d-glucose concomitantly causes a prolonged elevation of [Ca^2+^]_mito_ upon stimulation with histamine [110], which might occur either due to functional disconnection of fragmented mitochondria from the ER or due to inhibition of mitochondrial NCX by ROS [73] (Fig. 3). Persistent redistribution of Ca^2+^ between subcellular compartments, in turn, can initiate unfolded protein response [26] and apoptosis [61] both of which are observed in endothelial damage [24].

An enhancement of certain apoptotic pathways is seen in cellular models of diabetic vascular disease [42] that might also involve a caspase-independent/AIF-dependent pathway [83]. Other studies have shown that the Ca^2+^-dependent opening of mPTP participates in the death of human aortic endothelial cells under hyperglycemic conditions [119].

From a therapeutic point of view, the antidiabetic agent metformin is of special interest because in addition to its metabolic effects, it prevents the opening of mPTP [31]. This might contribute to its effectiveness in preventing cardiovascular endpoints [43]. Moreover, in hyperglycemia, as in hyperlipidemia, mitochondrial oxidative stress can be attenuated by treatment with resveratrol [141], probably through induction of antioxidative defense mechanisms within the cell [140].

### Lipotoxicity

Specific familial disorders and metabolic diseases such as type 2 diabetes can cause a mismatch between lipid supply and uptake capacity of the adipose tissue leading to increasing levels of free fatty acids, triglycerides and cholesterol in the blood plasma. Since the endothelium is not able to store large amounts of lipids, pathologically elevated blood lipids represent a tremendous metabolic challenge for endothelial mitochondria and a threat to the organelle’s function and integrity [53].

Proteins that are upregulated when fatty acid availability exceeds mitochondrial oxidative capacity are UCP2 and 3 [66, 67]. UCP3 was initially proposed to be a skeletal muscle isoform of the uncoupling protein family, responsible for mild uncoupling and reduction of ROS. However, UCP3 as well as UCP2 were also found to be present in endothelial mitochondria where they integrate a broad range of signaling functions including mitochondrial Ca^2+^ sequestration [55, 138, 145]. Beyond that, UCP2/3 were postulated to take part in the export of fatty acid anions that can accumulate in the mitochondrial matrix during lipid overload, hence preventing them from peroxidation [129]. Another group hypothesized that UCPs protect mitochondria from lipid-induced damage rather by removing lipid peroxides from the matrix [50]. In any case, knockdown of UCP2 in high fat fed mice causes endothelial dysfunction and aggravation of atherosclerosis [101], whereas its overexpression decreases ROS production and endothelin-1 gene expression while increasing eNOS levels [84]. Though the molecular functions of UCP2/3 are still a matter of debate, these proteins might represent promising candidates for therapeutic interventions in endothelial dysfunction caused by hyperlipidemia.

### Ischemia and reperfusion injury

Compared to other cell types such as neurons or the myocardium, the endothelium can tolerate long periods of ischemia [77]. In view of their primarily anaerobic energy metabolism [118], endothelial cells meet their energy demands even under severe hypoxic conditions of less than 0.1 mmHg pO_2_ [97]. This is probably supported by further restriction of mitochondrial oxidative metabolism through a Ca^2+^- and NO-mediated decrease in oxygen sensitivity of cytochrome c oxidase [25] that, in turn, preserves O_2_ levels for surrounding aerobic tissue that crucially depends on sufficient oxygen supply.

However, in contrast to their high resistance to ischemia, endothelial cells seem to be particularly vulnerable during reperfusion, as they are the first to undergo apoptosis starting already after 5 min of reperfusion in isolated rat hearts [126]. The importance of endothelial mitochondria in this process is highlighted by a report demonstrating that reperfusion injury in endothelial cells is mediated mainly via activation of caspase 9. This points towards mitochondrial damage, whereas apoptosis of cardiomyocytes was characterized by activation of caspase 8 [127]. So, what is it that damages endothelial mitochondria not during ischemia, but rather after reperfusion?

The most widely discussed factor that is proposed to damage endothelial mitochondria exclusively upon reperfusion are ROS, which can originate from numerous sources during reperfusion. The three most studied sources comprise the enzymes xanthine oxidase, NADPH oxidase and, of course, the mitochondrial ETC [113, 161]. While NADPH oxidase is mainly found in inflammatory cells, which can infiltrate the damaged tissue in large numbers after restoration of blood flow, both xanthine oxidase and mitochondria have been shown to contribute to postischemic ROS generation within the endothelium [11, 57, 113, 120]. Interestingly, transgenic mice overexpressing cytosolic CuZn–SOD show superior protection against endothelial dysfunction after reperfusion [68].

Repeated short episodes of ischemia, so-called ischemic preconditioning, improve cell survival after subsequent severe ischemia not only in cardiac muscle but also in the endothelium [82]. This beneficial effect appears to be, at least in part, mediated via ROS signaling. Free radical scavengers, on the one hand, have protective properties when administered during ischemia but, on the other hand, can abrogate the positive effects of preconditioning [10]. It is still not known in how far mitochondria contribute to preconditioning in the endothelium, but considering their central role in redox signaling and expression of adhesion molecules [1], all of which are affected during reperfusion [82], it is tempting to speculate that mitochondria play a central role in reperfusion injury and might represent promising targets for new therapeutic approaches. Especially attractive as potential drug targets would be mitochondrial Ca^2+^ channels. The significance of mitochondrial Ca^2+^ in endothelial reperfusion injury still requires further investigation. One can expect, though, that cytosolic Ca^2+^ oscillations occurring during reoxygenation [70] are transferred into the mitochondria where they may trigger ROS production [20] and exocytosis of adhesion molecules [71] that, in turn, favor reperfusion-associated endothelial dysfunction and leukocyte infiltration.

## Conclusion

Compared to other tissues, the endothelium has only few mitochondria and their contribution to cellular energy production is rather insignificant. Yet these organelles are of central importance to endothelial function as they integrate a broad spectrum of physiological processes including Ca^2+^ handling, redox signaling, mechanotransduction and apoptosis, all of which are closely interrelated. Dysfunction of endothelial mitochondria is considered to be a causative factor in the pathophysiology of most cardiovascular diseases and, thus, represents a promising target for future therapeutic interventions. However, the complexity of mitochondrial signaling pathways and interorganelle crosstalk is generally underestimated and requires further studies in order to be profoundly understood.

## Electronic supplementary material

Below is the link to the electronic supplementary material.

Supplementary movie 1 (MPG 2186 kb)

Supplementary movie 2 (MPG 3442 kb)
